# Suture Button with Tension Band Fixation for Patella fractures: A Retrospective Case Series

**DOI:** 10.51894/001c.141751

**Published:** 2025-08-07

**Authors:** Jacob Archutowski, Saif Juma, William C. Searls, Tyson Roderique, Vinay Pampati

**Affiliations:** 1 Henry Ford Macomb Hospital, Clinton Twp, MI; 2 College of Osteopathic Medicine Michigan State University, East Lansing, MI

**Keywords:** Patella fracture, button, fixation, surgery

## Abstract

**INTRODUCTION:**

There is a high rate of surgical complications and removal of symptomatic hardware for patients who have undergone open reduction internal fixation (ORIF) for transverse patella fractures. In recent years there has been increased interest in more low profile ORIF techniques to combat these issues. The aim of this study was to evaluate if a reduced hardware burden would correlate with fewer complications and equal rates of fracture union when compared to traditional techniques for treating transverse patella fractures.

**METHODS:**

Nine patient charts were retrospectively reviewed dating between June 2015 and March 2023. All patients sustained a transverse patella fracture and underwent ORIF with a suture button and suture tension band construct by a single surgeon. The primary outcome measure was rate of radiographic fracture union at final follow up. Secondary outcome measures included any need for removal of hardware or other revision procedure, surgical and medical complications, postoperative pain score and the ability to perform a straight leg raise.

**RESULTS:**

Eight of nine patients demonstrated radiographic evidence of fracture consolidation with an average follow-up time of 17.9 months (range 12-26 months). One patient required an additional operation for revision ORIF before going on to successful union. No patients underwent a procedure for removal of hardware before final follow up. All patients were able to hold a straight leg raise at final follow up.

**CONCLUSIONS:**

Suture button with suture tension band construct is a reasonable treatment option for treating transverse patella fractures. Surgeons may employ this technique for older patients or those with some fracture comminution, although there should be some caution and close follow up for displacement.

## INTRODUCTION

Patella fractures account for one percent of all skeletal injuries. Indications for surgical treatment include an incompetent extensor mechanism, fracture separation greater than 2-4 mm, step-off greater than 2-3 mm, and intra-articular loose bodies.^1^ Surgical treatment options include open reduction internal fixation (ORIF) and partial or total patellectomy. Patellectomy is reserved for cases in which ORIF is not possible, as it is associated with significant decrease in extensor mechanism strength.

Various ORIF techniques exist, including the use of a tension band constructs with K-wires, cannulated screws, mini-fragment or mesh plates and cerclage wiring. ORIF, regardless of technique, is reported to have >95% rate of union.^2^ However, symptomatic hardware after patella ORIF requiring removal is common, with re-operation rates due to symptomatic hardware reported up to 33.6%.^2^ Checketts et al report only 72.4% of patients who underwent patella ORIF had minimal to no symptoms at one year post-operatively, leaving 27.6% symptomatic and 9.4% opting to undergo revision surgery.^3^

Over the last decade, there has been a surge in literature exploring hybrid or all suture fixation methods for fixating patella fractures in an attempt to combat these complications. Gosal et al compared rates of complications using metal wire and non-absorbable polyester for patella fracture fixation.^4^ Reoperation rates when metal wire was used was 38% compared to 6% when nonabsorbable polyester was used.

In 2017 Han et al performed a biomechanical cadaveric study comparing a double button adjustable loop device versus a tension band wire for fixating transverse patella fractures.^5^ They found there was no difference in fracture gapping between the two groups when loaded 500 times. This technique was also described in a small case series of young athletes by Bukva et al.^6^ They utilized an Arthrex TightRope™, most commonly used for ankle syndesmotic fixation, and concluded that using suture button fixation in combination with braided polyester in a tension band construct not only achieved fracture healing but also has a low risk of complication and no need for hardware removal.^6^ The purpose of our study was to examine the efficacy of suture button fixation for the treatment of transverse patella fractures. Our hypothesis was to see an equivalent rate of fracture union when compared to more traditional surgical techniques, with a decreased rate of surgical complications and removal of symptomatic hardware.

## METHODS

The authors obtained approval of their protocol from their Institutional Review Board prior to any data collection. A retrospective case series of nine patients was conducted between 2015 and 2023. All patients were treated surgically by a single fellowship-trained Orthopaedic Sports Medicine surgeon.

Patients were included for analysis if they had undergone ORIF of a transverse patella fracture utilizing suture buttons with the addition of a figure-of-eight braided polyester and ultra-high molecular weight polyethylene suture tension-band, and a minimum of twelve months follow up. Patients were excluded from analysis if they were under age 18, had less than twelve months follow up or any prior surgical intervention for the fracture being treated.

Upon presentation, all patients were unable to perform a straight leg raise and their radiographs demonstrated transverse patella fractures. Although some of our patients had fracture comminution which required a more tedious reduction, they received no additional fixation. All patients were treated with the same procedural steps described below, with no remarkable deviations in technique. In all cases, Arthrex TightRopes^TM^ were used for suture button fixation. A size 5 Arthrex SutureTape^TM^ was used for anterior tension band in all but one case, where an Arthrex FiberWire^TM^ was used (Patient 1).

### Procedural Technique

**Figure 1.**
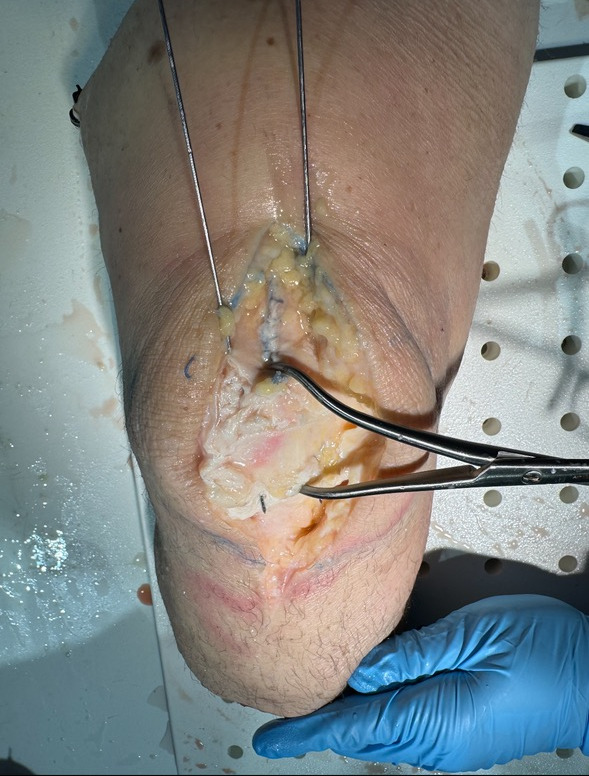
A reduction is placed across the fracture site followed by two 1.6 mm pins which will guide the cannulated drill bit.

**Figure 2.**
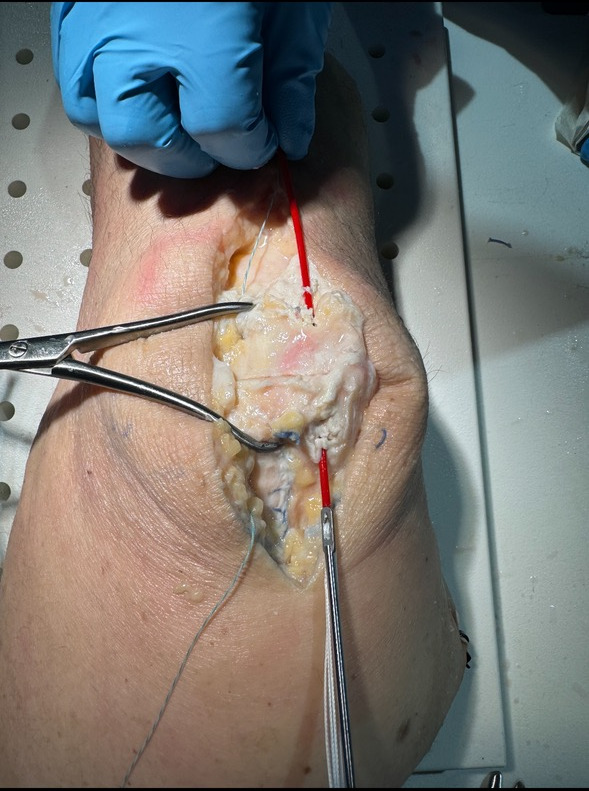
An Arthrex TightRope is shuttled across the fracture site.

All patients were placed in the supine position with a tourniquet on the upper thigh, which was inflated prior to incision. An incision was made centered over the patella and dissection was carried down to the fracture site with care to avoid damage to the quadriceps and patellar tendons. After irrigation of the fragments, fluoroscopy was utilized to ensure proper reduction with clamps. Medial and lateral K-wires were then driven across the fracture in a perpendicular fashion (Figure 1). Proper placement was ensured via fluoroscopy. A small incision was then made through the quadriceps tendon to create room for a 3.5 mm cannulated overdrill along the K-wire. This step was repeated on the patellar tendon site. This allowed passage of our suture buttons (Arthrex TightRope^TM^) through each drill hole with a suture passer. At this point, buttons were then present in all four quadrants of the patella (Figure 2). Minimal tensioning of the suture buttons was then performed.

**Figure 3.**
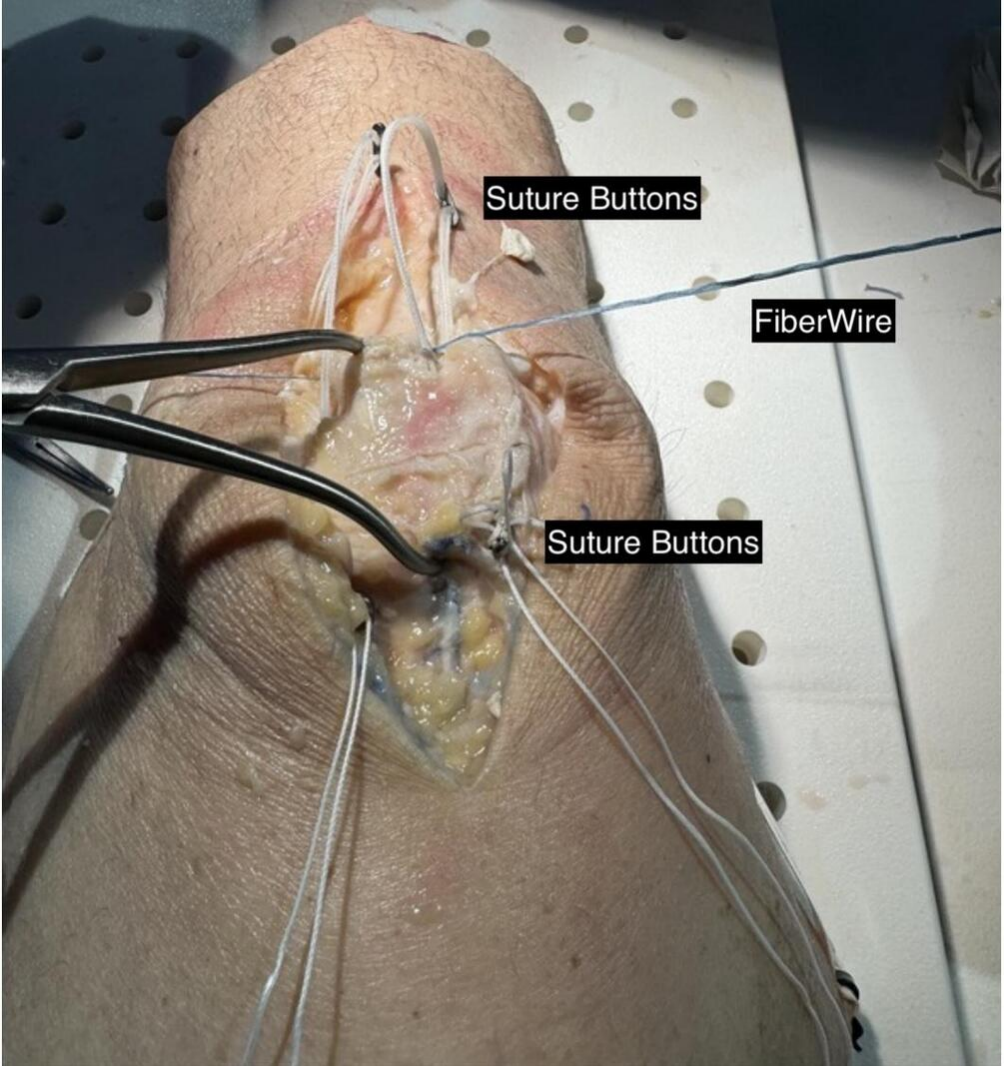
A #2 FiberWire is placed through the loops of the TightRope in a figure-of-8 manner.

A braided polyester and ultra-high molecular weight polyethylene suture (Arthrex FiberWire or SutureTape^TM^) was then placed over the suture button ends in a figure-of-eight manner (Figure 3). Suture buttons were each sequentially tensioned with the knee in full extension, which secured the FiberWire/Suture Tape to bone. Finally the figure-of-eight suture would be tied to complete the anterior tension-band.

**Figure 4.**
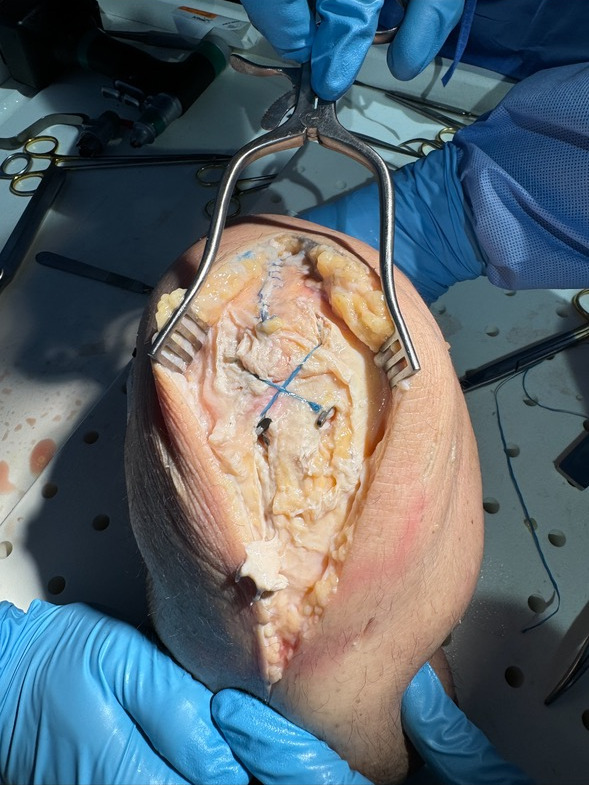
Intraoperative demonstration of the finished double suture button fixation with anterior suture tension band

**Figure 5.**
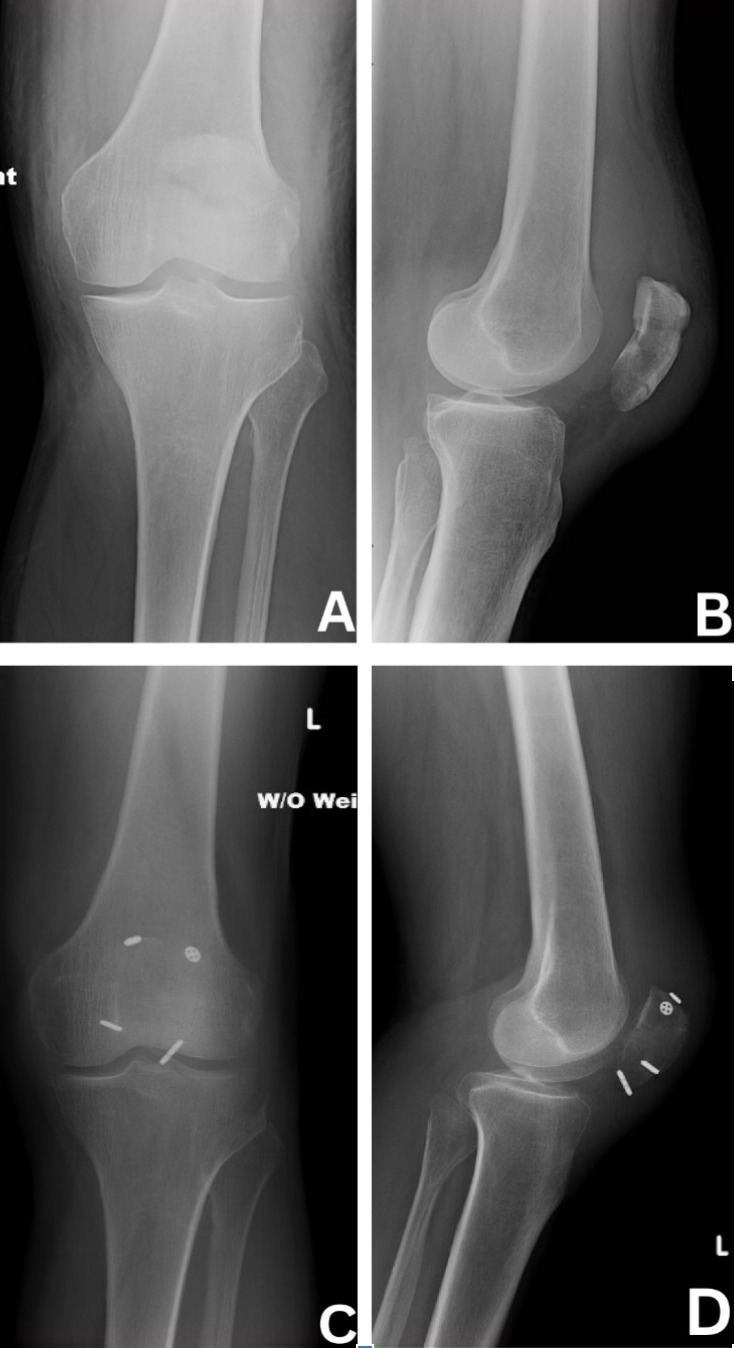
A and B demonstrate preoperative radiographs of a transverse patella fracture. C and D are postoperative radiographs of successful fixation of the fracture after ORIF using our suture button and suture tension band construct.

Fluoroscopy was used to confirm appropriate reduction of the articular surface (Figure 5). The knee was then taken through range of motion to ensure there was no gapping while taking care not to compromise the repair. The wound was copiously irrigated and closed with close inspection of the retinaculum. If the retinaculum was disrupted, it was then repaired with a combination of SutureTape and Vicryl suture.

### Postoperative Protocol

All nine patients followed the same postoperative protocol. They were seen at two, six and twelve weeks for a wound check, and to record their range of motion as well as a rating of pain on a subjective 0-10 scale. Radiographs were obtained at each postoperative visit until fracture union was demonstrated; we define radiographic union as expected callous formation with resolution of fracture lines. In the immediate postoperative period, patients were placed in a hinged knee brace locked in extension and allowed to weight bear as tolerated on the operative extremity. At the initial two-week follow up visit, patients progressed to 0-30 degrees in the hinged knee brace and were able to progress through an additional 15 degrees of flexion each subsequent week. No active knee extension was recommended from the initial postoperative period to six weeks. At six weeks, patients received formal physical therapy with instructions for focus on isometric quadriceps strengthening. Patients and physical therapists were asked to avoid plyometric exercises for a minimum of twelve weeks postoperatively. There was no documentation of patients being non compliant or deviating from our protocol.

## RESULTS

The average patient age was 50.8 years (range, 20–72). Average follow-up of 17.9 months (range: 12–26 months). The mechanism of injury was similar for most patients: six occurred from a ground level fall, two occurred from fall from height, and one occurred following a motorcycle crash. Eight of nine (89%) patients demonstrated radiographic evidence of fracture consolidation following the index procedure. Patient 6 experienced fracture displacement at the first follow-up which required a revision ORIF using a star plate before going on to successful union. This was the only surgical or medical complication encountered, accounting for an 11 % complication rate (Table 1).

**Table 1.** Patient Demographics and Postoperative outcomes measures at final follow up

**Patient**	**Age**	**Sex**	**Fracture comminution**	**Radiographic union**	ROM*	**Pain** **Score/10**	**Extensor Mechanism Intact**	**Complication**	**Secondary Procedure**
**1**	63	M	No	Yes	10-140	0	Yes	No	No
**2**	56	M	No	Yes	0-120	3	Yes	No	No
**3**	21	F	Yes	Yes	0-140	5	Yes	No	No
**4**	28	M	No	Yes	0-130	4	Yes	No	No
**5**	63	F	Yes	Yes	0-130	0	Yes	No	No
**6**	67	F	Yes	No**	10-130	3	Yes	Fracture Displacement	Revision ORIF
**7**	20	M	Yes	Yes	0-130	6	Yes	No	No
**8**	72	F	No	Yes	0-120	7	Yes	No	No
**9**	67	F	No	Yes	0-120	2	Yes	No	No

*ROM: Range of Motion

**Patient 6 achieved radiographic union at final follow up following revision ORIF

All patients were able to perform a straight leg raise at final follow-up. The majority of patients reported some degree of postoperative pain. The median postoperative pain score was 3/10, with a range from 0-7/10 at final follow up. Postoperative range of motion (ROM) was recorded in all patients, with most achieving 0°–130° flexion. One patient exhibited mild extension lag at final follow-up. The extensor mechanism remained intact in all patients.

A few patients had some form of postoperative abnormality, including pain, swelling, or a positive patellar grind test in one case. However, no cases of infection, hardware failure, or other major surgical complications were observed. None of the patients have required removal of the suture button or tension band construct for reasons of symptomatic hardware or pain.

## DISCUSSION

Transverse patella fractures can be treated operatively via a number of different techniques depending on the patient and fracture characteristics. Traditional operative techniques include ORIF via plating, tension banding or cannulated screws. These options have shown to have a high complication and hardware removal rate.^2^ There have also been rare but serious complications reported such as migration of broken K-wires to the popliteal fossa.^7^ In recent years, there has been a movement towards more low profile suture based implants for patella ORIF to combat these complications.^8^ The purpose of our study was to assess the rate of union, complications and removal of hardware rate of a suture and button based technique implemented for our patient cohort. Our hypothesis was to see a rate of union equivocal to that of traditional patella ORIF, with a decreased rate of hardware removal or hardware related complications.

Eight of our nine patients included in the study went on to fracture union following the index procedure. All patients were able perform a straight leg raise, and had nearly full range of motion. To our knowledge, none of our cohort underwent secondary procedure for hardware removal or complained of symptomatic hardware. This is lower than widely reported rates of symptomatic hardware, and surgical complications of more traditional techniques, which have reported reoperation rates up 40% .^2,9^ Of note, although one of our patients experienced a mechanical failure of the implant resulting in fracture displacement, we do not include this as a repeat surgery for symptomatic hardware removal.

Our results are comparable to more traditional techniques for patella ORIF with larger hardware burden. Busel et al saw 48 of their 50 patients go on to union following a similar technique for patella ORIF using suture tension banding combined with cannulated screws across the fracture site. Average time to union was 3.1 months. However they saw an eight percent hardware removal rate.^10^

Sun et al implemented a similar technique for three inferior pole patella fractures using double button plates. At twelve months follow up, all of their cohort had gone on to heal, with no noted complications or revision surgeries.^11^ Chen et al compared a matched cohort of 50 patients who underwent patella ORIF with K wire tension banding versus a transosseous suture technique. They found no significant difference in time to union between the two groups. In addition they saw significantly more reoperations in the tension band group as well as the complication of skin irritation.^12^

Limited reports have previously described our technique.^5,6^ However, this study would be the largest and most age-diverse cohort analyzed. Bukva et al implemented the same tension band suture and button technique for four athletes with an average age of 26, all of whom sustained transverse patella fractures.^6^ All four of their patients went on to radiographic union by three months, with no reported hardware removal at final follow up. In contrast to their study, our patient sample included an almost bimodal distribution of younger and older patients, with three below age 30 and six above age 50. Unsurprisingly, the older subcohort of patients more often had comminution in their fractures and gave rise to our only complication which was failure of fixation and displacement. In the case of patient 6, there were no technical issues noted in the operating room, nor documented issues with protocol compliance. We suspect a combination of decreased bone quality and fracture comminution as the most likely cause of failure.

Failure of a similar technique using button plates has been described when used in a comminuted fracture.^13^ Additionally, in a 2012 study by Miller et al, they found age to be a predictor of fixation failure after patella ORIF regardless of technique.^14^ Taking our complication and the literature into consideration, we recommend close postoperative radiographic follow up after implementing this technique for older patients with expected poor bone quality or more comminuted fractures.

There are several limitations to this study. First, this is a retrospective review with no control group. Second, our study is limited to a small sample of nine patients, with virtually no statistical power to detect outcome differences. Third, some patients had only short term follow up, it is unknown if they sought out a different surgeon outside our records. Further larger cohort studies with control group comparison and longer term follow up are needed. Future research may include a cost analysis, as we feel this would be valuable information given that patients treated with ORIF techniques containing metal implants are more likely to require the added cost of a second surgery.

## CONCLUSION

Suture button fixation with suture tension banding is a reasonable alternative to treat transverse patella fractures. Our study demonstrates equivocal rates of fracture healing with fewer removal of hardware surgeries and complications than what is reported for traditional techniques. Surgeons should observe some caution when employing this technique if bone quality or fracture comminution is a concern.

### Conflict of Interest

None.
